# Accelerated epigenetic aging and mitochondrial DNA copy number in bipolar disorder

**DOI:** 10.1038/s41398-017-0048-8

**Published:** 2017-12-11

**Authors:** Gabriel R. Fries, Isabelle E. Bauer, Giselli Scaini, Mon-Ju Wu, Iram F. Kazimi, Samira S. Valvassori, Giovana Zunta-Soares, Consuelo Walss-Bass, Jair C. Soares, Joao Quevedo

**Affiliations:** 1 Translational Psychiatry Program, Department of Psychiatry and Behavioral Sciences, McGovern Medical School, University of Texas Health Science Center at Houston (UTHealth), 1941 East Rd, 77054 Houston, TX USA; 2 Center of Excellence on Mood Disorders, Department of Psychiatry and Behavioral Sciences, McGovern Medical School, The University of Texas Health Science Center at Houston (UTHealth), Houston, TX USA; 3 Laboratory of Neurosciences, Graduate Program in Health Sciences, Health Sciences Unit, University of Southern Santa Catarina (UNESC), Criciúma, SC Brazil; 4 Neuroscience Graduate Program, The University of Texas MD Anderson Cancer Center UTHealth Graduate School of Biomedical Sciences, Houston, TX USA

## Abstract

Bipolar disorder (BD) has been previously associated with accelerated aging; yet, the mechanisms underlying this association are largely unknown. The epigenetic clock has been increasingly recognized as a valuable aging marker, although its association with other biological clocks in BD patients and high-risk subjects, such as telomere length and mitochondrial DNA (mtDNA) copy number, has never been investigated. We included 22 patients with BD I, 16 siblings of BD patients, and 20 healthy controls in this analysis. DNA was isolated from peripheral blood and interrogated for genome-wide DNA methylation, mtDNA copy number, and telomere length. DNA methylation age (DNAm age) and accelerated aging were calculated using the Horvath age estimation algorithm in blood and in postmortem brain from BD patients and nonpsychiatric controls using publicly available data. Older BD patients presented significantly accelerated epigenetic aging compared to controls, whereas no difference was detected among the younger subjects. Patients showed higher levels of mtDNA copy number, while no difference was found between controls and siblings. mtDNA significantly correlated with epigenetic age acceleration among older subjects, as well and with global functioning in our sample. Telomere length did not show significant differences between groups, nor did it correlate with epigenetic aging or mtDNA copy number. These results suggest that BD may involve an accelerated epigenetic aging, which might represent a novel target for treating BD and subjects at risk. In particular, our results suggest a complex interplay between biological clocks to determine the accelerated aging and its consequences in BD.

## Introduction

Accelerated aging is commonly observed in several chronic illnesses, increasing the rate of aging-related medical conditions and shortening the life span in patients^1–4^. In particular, bipolar disorder (BD), an often severe and highly disabling psychiatric condition that affects around 1% of the population^5^, has been characterized by many features of aging that can complicate disease outcomes and converge to premature aging (commonly described as “accelerated aging” in the field)^6,7^.

As a multidimensional construct, aging includes physical, psychosocial, and biological changes. The latter are commonly referred to as “senescence”, a concept traditionally used for individual cells to explain their limited capacity to proliferate, but that can also be applied to whole organisms^6^. The study of biological aging (i.e., the molecular mechanisms involved in the age-related decline in physiological functions and components of an organism^1^) in clinical populations generally relies on the measure of relevant “biological clocks”, among which telomere length has been the most commonly investigated. Specifically, studies of telomere length in BD have yielded contrasting results. The majority of findings showed telomere shortening^8–13^, while few others revealed either no significant differences compared to controls^14–16^ or a longer telomere length in BD patients possibly due to the effects of medications^17^. Interestingly, first-degree relatives of BD patients have been recently shown to present shorter telomeres compared to patients^18^ and healthy controls^13,18^, although multiple aging markers have yet to be measured in this high-risk population. Accordingly, data regarding telomeres in patients with severe mental illness are not robust enough to determine that they are a reliable marker of premature aging. Although several studies reported telomere shortening^19,20^, some actually found the opposite direction^21,22^, even after controlling for medication exposure^21^. This suggests that novel, more specific aging markers are needed, with a potential approach being an integrative analysis involving multiple markers.

In this sense, DNA methylation, an epigenetic marker associated with BD pathophysiology and treatment^23^, has been shown to accurately predict chronological age in multiple tissues^24^. This so-called “epigenetic aging” has been recently shown to be accelerated with cumulative lifetime stress^25^, and a faster-running epigenetic clock has been significantly linked to higher mortality risk^26^. Another potential biological clock is the number of copies of mitochondrial DNA (mtDNA), which is tightly associated with mitochondrial function. This marker is of relevance for biological aging as aging has been consistently associated with a progressive dysfunction in respiratory chain activity and cumulative mitochondrial dysfunction^27^. Accordingly, some studies have found significant correlations between mtDNA copy number and chronological age^28^, although not in all populations^29,30^. Importantly, while mtDNA copy number and telomere length have been significantly correlated^31–33^, recent studies suggest that DNA methylation age and telomere length are uncorrelated and independently predict chronological age^34,35^.

To date, no study has ever investigated the cross talk between these three biological clocks in the same clinical sample. Thus, this study aimed to assess these aging markers in patients with BD and siblings of BD patients, with the ultimate goal of identifying mechanisms involved in the accelerated aging observed in BD and its risk.

## Materials and Methods

### Sample

Twenty-two patients with BD I, 16 siblings of BD patients, and 20 healthy controls were enrolled for this study, all matched by age, sex, ethnicity, and race. Subjects were recruited at the University of Texas Health Science Center at Houston, where the local institutional review board approved the protocol. Informed consent was obtained from all participants upon enrollment and prior to any procedure. Participants had no current medical disorder including neurological disorders and traumatic brain injury, schizophrenia, developmental disorders, eating disorders, and intellectual disability. Siblings were enrolled provided that they had at least one relative who met the criteria for BD as determined via a detailed family history assessment, and 14 of them (87.5%) were related to the patients recruited in this study. Healthy controls were excluded if they had a history of any Axis I disorder in first-degree relatives or if they had taken a prescribed psychotropic medication at any point in their lives. Female participants of reproductive age underwent a urine pregnancy test. All participants underwent a urine drug screen to exclude illegal drug use.

### Clinical assessments

Psychiatric diagnosis of individuals with BD and their siblings was based on the Structured Clinical Interview for the DSM-IV Axis I Disorders (SCID I) interview. All interviews were administered to participants by trained evaluators and later reviewed by a board-certified psychiatrist. Individuals’ current affective state was assessed with the Young Mania Rating Scale (YMRS)^36^ and the Montgomery–Åsberg Depression Rating Scale (MADRS)^37^. Global functioning was assessed by the Global Assessment of Functioning Scale (GAF)^38^.

### Genome-wide methylation analysis

Peripheral blood was drawn from fasting subjects in EDTA-containing tubes, followed by DNA isolation from buffy coat with the DNeasy Blood & Tissue Mini Kit (Qiagen, Hilden, Germany), according to the manufacturer’s instructions. Isolated DNA samples were quantified on NanoDrop (Thermo, Waltham, MA, USA) and bisulfite-converted (500 ng) using the EZ DNA Methylation™ Kit (Zymo Research, Irvine, CA, USA), followed by hybridization on the Infinium HumanMethylation450 BeadChip Kit (Illumina, San Diego, CA, USA). Beadchips were scanned in an iScan microarray reader (Illumina), and analyses were performed using the RnBeads R package^39^. Details of the DNA methylation analyses can be found in the Supplementary Methods. Genome-wide DNA methylation levels measured in postmortem cerebellum tissue from BD patients (*n* = 47) and controls (*n* = 47) were also extracted from a publicly available data set (GSE38873)^40^ at the Gene Expression Omnibus (GEO) platform (ncbi.nlm.nih.gov/geo/) for further analyses, as described below.

### Epigenetic clock

The DNA methylation age (DNAm age) was calculated in blood samples and in the data from GSE38873 using the Horvath age estimation algorithm^24^ with a freely available online tool (http://labs.genetics.ucla.edu/horvath/htdocs/dnamage/). The algorithm predicts DNAm age based on the methylation levels of 353 CpGs from the Illumina 450 K Beadchip^24^ and is applicable to all types of tissues and cells. The online tool uses an elastic net-penalized regression model (implemented in the R package) and provides an estimate of accelerated epigenetic aging (in years) by regressing DNAm age on chronological age, forming residuals that can be compared between groups.

### mtDNA copy number and telomere length

Real-time quantitative polymerase chain reactions (PCRs) were performed to measure the amount of mitochondrial DNA with a modified protocol from Tyrka and collaborators^41^, and telomere length as previously described^42^. Please see Supplementary Methods for more details.

### Statistical analysis

Categorical variables were compared between groups with chi-square tests. Parametric distribution of the continuous variables was tested by Shapiro–Wilk test and histogram visualization. Age, years of education, body mass index, GAF, and cell count estimates (monocytes and CD4 + T-lymphocytes) were analyzed with one-way analysis of variance (ANOVA) followed by post hoc test of Tukey, when appropriate, with a *p*-value < 0.05 deemed as significant. MADRS, YMRS, and cell count estimates (B-lymphocytes, granulocytes, natural killer cells, and CD8 + T-lymphocytes) showed nonparametric distributions and were therefore analyzed by Kruskal–Wallis tests followed by Mann–Whitney tests with a Bonferroni-corrected *p*-value < 0.016 deemed as significant. Epigenetic aging acceleration (measured in blood), mtDNA copy number, and telomere length were compared between patients, siblings, and controls by linear regression models using chronological age, sex, and cell count estimates (monocytes, CD4 + T-lymphocytes, B-lymphocytes, granulocytes, natural killer cells, and CD8 + T-lymphocytes) as covariates. Epigenetic aging acceleration in cerebellum samples was compared between BD patients and nonpsychiatric controls by a linear regression model controlling for postmortem interval (PMI). Correlations between variables were tested with either Pearson or Spearman tests, depending on their distribution.

## Results

### Sample

Controls, BD patients, and siblings showed no differences for age, sex, ethnicity, race, years of education, smoking status, or body mass index (Table 1). Chronological age ranged between 22 (youngest) and 51 (oldest) years old among controls, 20 and 51 among patients, and 19 and 58 among siblings (*p* = 0.325). Patients showed significantly higher scores on the MADRS and YMRS compared to controls (although of mild severity, attesting to their euthymic status), as well as lower functioning scores, as assessed by the GAF compared to siblings and controls. In total 10 out of the 16 siblings enrolled were asymptomatic (unaffected) and presented with no current or lifetime history of mental illness. The other six were diagnosed with alcohol use disorder (*n* = 3), generalized anxiety disorder (*n* = 1), specific phobia (*n* = 1), and posttraumatic stress disorder (*n* = 1). Seventy-two percent of the patients were on medications (Table 1), while none of the siblings or controls were medicated at the time of enrollment.

**Table 1:** Sample demographics

	Controls (*n* = 20)	BD I (*n* = 22)	Siblings (*n* = 16)	*P*-value
Age (years), mean ± SD	34.75 ± 10.0	33.95 ± 9.3	39 ± 10.6	0.325^a^
Sex (F/M)	12/8	15/7	10/6	0.852^b^
Ethnicity (H/N)	6/14	4/18	4/12	0.668^b^
Race (H/AA/W/A)	6/7/5/2	4/8/9/1	5/6/5/0	0.757^b^
Education (years), mean ± SD	15.22 ± 1.6	14.10 ± 2.2	15.0 ± 2.1	0.198^a^
Smoking (%)	11.1	30	30	0.321^b^
Body mass index (kg/m^2^), mean ± SD	28.18 ± 4.7	30.08 ± 7.9	28.88 ± 6.1	0.626^a^
MADRS, median (IQR)	0 (0)	8.5 (20)	1 (2)	< 0.001^c#^
YMRS, median (IQR)	0 (0)	5 (9)	1 (2)	< 0.001^c#^
GAF, mean ± SD	90.44 ± 4.4	61.82 ± 11.7	89.80 ± 4.4	< 0.001^a#†^
Age of onset (years)		19.45 (5.7)		
Length of illness (years)		13.8 (8.6)		
Number of episodes (%)				
0–3		18.1		
4–9		9.1		
> 10		72.7		
Medication use (%)				
Lithium		23.8		
Anticonvulsants		23.8		
Antidepressants		27.2		
Atypical antipsychotics		45.4		
Typical antipsychotics		4.7		
Benzodiazepines		13.6		
Comorbidities (%)				
GAD		27.2	6.2	
PTSD		13.6	6.2	
Social phobia		27.2	0	
PD		31.8	0	
Agoraphobia		13.6	0	
Bulimia		13.6	0	
BED		13.6	0	
AUD		0	18.7	

*A* Asian, *AA* African American, *AUD* alcohol use disorder, *BED* binge-eating disorder, *F* female, *GAD* generalized anxiety disorder, *GAF* Global Assessment of Functioning scale, *H* Hispanic or latino, *IQR* interquartile range, *N* Non-Hispanic or latino, *PD* panic disorder, *PTSD* posttraumatic stress disorder, *W* White or Caucasian.

^a^One-way ANOVA;

^b^chi-square test;

^c^Kruskal–Wallis test

^#^Different between BD patients and controls

^†^Different between siblings and BD patients.

### Genome-wide methylation analysis

We initially performed a preliminary methylome analysis looking for differences between controls, BD patients, and siblings in individual CpG probes and regions. No locus or regions withstood false-discovery rate correction for multiple testing (adjusted *p*-value > 0.05 for all comparisons), possibly due to a lack of statistical power. Considering the cell-specific patterns of methylation, we also used the methylation data to predict the cell-type composition in blood. No differences were found in the predicted frequency of B-lymphocytes, granulocytes, monocytes, natural killer cells, CD4^+^ T-lymphocytes, or CD8^+^ T-lymphocytes between groups (Supplementary Table S1).

### Epigenetic clock in blood

**Fig. 1:**
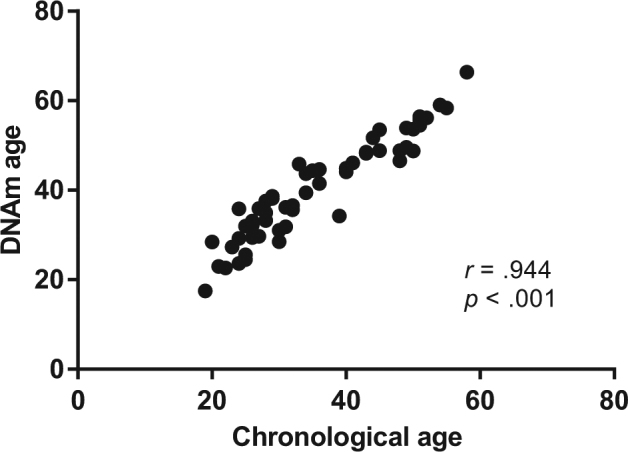
Scatterplot illustrating the significant and positive correlation between DNA methylation age (DNAm age in years, calculated based on the Horvath algorithm) and chronological age (years). Analysis was performed by Pearson correlation coefficient

**Fig. 2:**
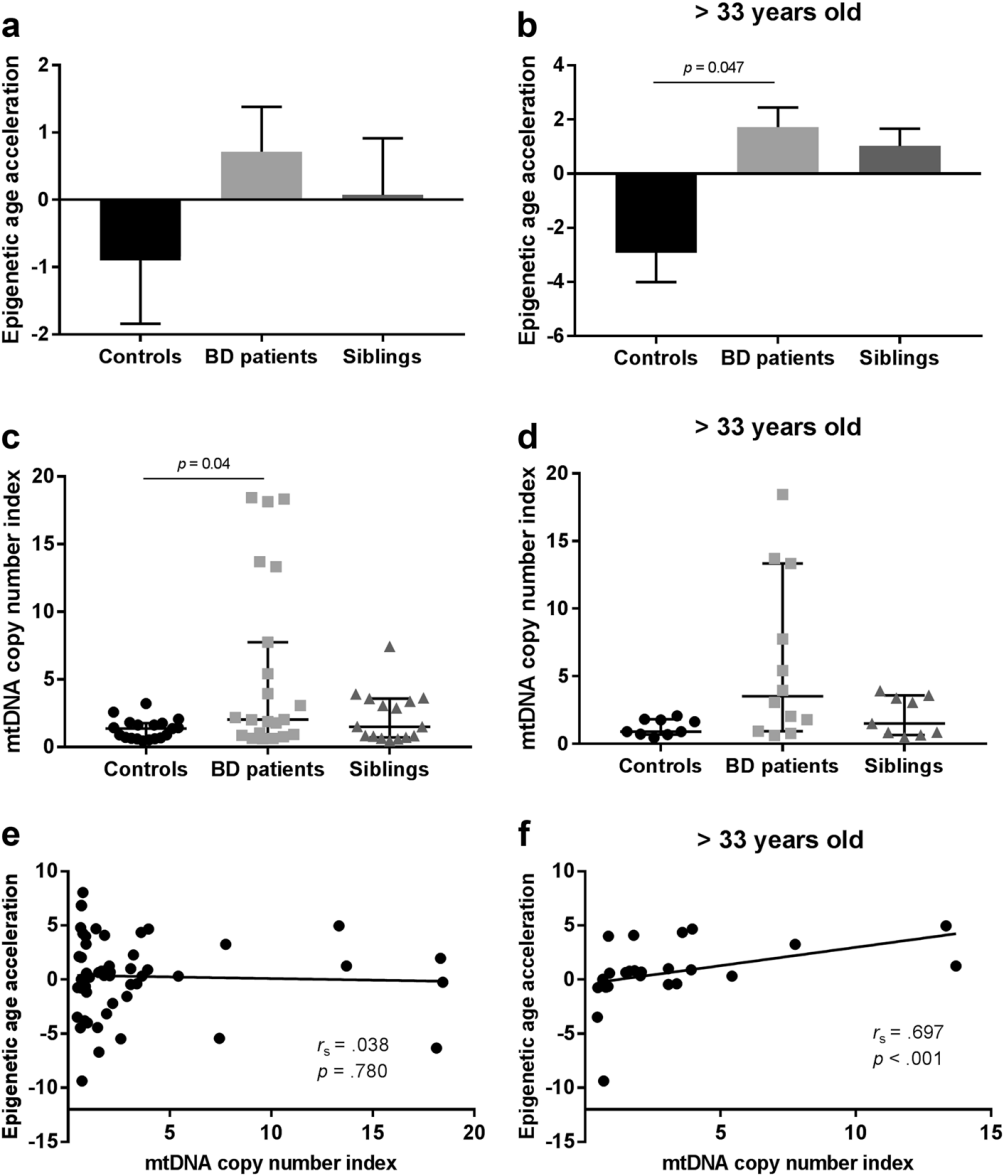
Epigenetic age acceleration and mitochondrial DNA (mtDNA) copy number in patients with bipolar disorder and siblings Bars (panels **a** and **b**) represent mean + standard error of the mean. Epigenetic age acceleration was calculated by regressing the predicted DNA methylation (DNAm) age to the chronological age of the subjects. Negative and positive values represent younger and older DNAm ages compared to their chronological ages, respectively. Panels **c** and **d** represent median ± 95% confidence interval of mtDNA copy number in each group. Panels **a**, **c**, and **e** represent the analyses performed with the whole sample, while panels **b**, **d**, and **f** represent only the older subjects ( > 33 years). **a** Between-group comparison of epigenetic age acceleration in the entire samples (*n* = 58). **b** Between-group comparison of epigenetic age acceleration in subjects older than 33 years old. **c** mtDNA copy number in the entire sample. **d** mtDNA copy number in subjects older than 33 years old. **e** Correlation between epigenetic age acceleration and mtDNA copy number in the entire sample. **f** Correlation between epigenetic age acceleration and mtDNA copy number in subjects older than 33 years old

DNAm age and a measure of accelerated epigenetic aging were calculated for each individual based on the 450K data. As expected, there was a strong positive correlation between individuals’ DNAm age and chronological age (*r* = 0.944, *p* < 0.001; Fig. 1). Accelerated epigenetic aging did not differ between men and women, either when analyzing the entire sample or when checking for differences within each diagnosis group (*p* < 0.05). When analyzing the entire sample, accelerated epigenetic aging showed no difference between groups after controlling for chronological age, sex, and blood cell-type composition (*F*(2, 46) = 2.043, *p* = 0.141; η^2^_p_ = 0.082) (Fig. 2a). Nonetheless, based on previous findings showing a cumulative effect of lifetime stress on epigenetic aging^25^, we sought to investigate whether a difference would be detected when limiting our analysis specifically to older subjects. We therefore split our sample into two smaller groups of younger and older subjects based on the median of the chronological age (33 years old) and performed our analyses a second time. As hypothesized, accelerated epigenetic aging was significantly different between groups in the older subsample (*F*(2, 17) = 3.924, *p* = 0.04, and η^2^_p_ = 0.316, Fig. 2b), with BD patients presenting a higher accelerated aging compared to controls (pairwise comparison’s Bonferroni-corrected *p* = 0.047). Siblings did not significantly differ from controls (*p* = 0.159). No difference between groups was detected among the younger subjects (*F*(2,18) = 0.095, *p* = 0.910, and η^2^_p_ = 0.010).

### Validation in brain tissue

We used publicly available DNA methylation data from postmortem cerebellum samples from BD patients and controls (GSE38873)^40^ to check whether accelerated epigenetic aging would be detected in brain tissue, as well. Specifically, several cerebellar changes have been reported in patients with BD, including decreased cerebellar volume, cerebellar atrophy^43^, aberrant cerebellar connectivity^44^, and microstructure abnormalities^45^. Age, DNAm age, and postmortem interval (PMI) of the samples analyzed are shown in Supplementary Table S2. As expected, DNAm age and chronological age were significantly correlated (*r* = 0.830, *p* < 0.001, Supplementary Fig. S1A). We found no statistically significant difference in accelerated epigenetic aging between patients and controls when analyzing the entire sample and controlling for PMI (*F*(1, 91) = 1.854, *p* = 0.177, and η^2^_p_ = 0.020; Supplementary Fig. S1B). We further split the sample into younger and older groups based on the median age (45 years old) and reran the analysis between groups, as similarly performed with the blood samples, but no significant differences were found within the younger (*F*(1, 34) = 0.063, *p* = 0.803, and η^2^_p_ = 0.002, Supplementary Fig. S1C) or older individuals (*F*(1, 54) = 2.894, *p* = 0.095, and η^2^_p_ = 0.051, Supplementary Fig. S1D).

### mtDNA copy number

No correlation was observed between mtDNA copy number and chronological age (*r*_s_ = 0.054, *p* = 0.693), and a significantly higher mtDNA copy number index was observed in women compared to men (U = 172, *p* = 0.003). When analyzing the entire sample controlling for chronological age, sex, and cell count estimates, mtDNA copy number was significantly different between groups (*F*(2, 43) = 3.963, *p* = 0.026, and η^2^_p_ = 0.156, Fig. 2c). Specifically, BD patients presented a higher mtDNA copy number index compared to controls (*p* = 0.04), while controls and siblings did not show any difference (*p* > 0.05). When we compared mtDNA copy number within younger and older subjects (median 33 years old, as performed for the epigenetic clock analysis), we found no significant differences between groups (younger subjects—*F*(2, 18) = 0.845, *p* = 0.446, and η^2^_p_ = 0.086; older subjects— F(2, 14) = 3.570, *p* = 0.056, and η^2^_p_ = 0.338).

### Association between epigenetic clock and mtDNA copy number

Although mtDNA copy number and the accelerated epigenetic aging score were not significantly correlated when considering the entire sample (*r*_s_ = 0.038, *p* = 0.780, Fig. 2e), they were positively correlated within the older group (*r*_s_ = 0.697, *p* < 0.001, Fig. 2f). As an exploratory analysis to further explore this association and based on previous evidence of DNA methylation control of nuclear-encoded mitochondrial genes^46^ (i.e., defined as genes coding for mitochondrial proteins that are encoded in the nuclear DNA), we checked whether some of the genes that compose the epigenetic clock included mitochondrial genes. Our analyses showed that out of the 353 clock CpGs^24^, 17 of them are located within nuclear-encoded mitochondrial genes (according to the latest update on the Human MitoCarta2.0 data set^47^) (Table S3). Further details of these genes, including their exact genomic location and annotation, can be found in Supplementary Table S3.

### Telomere length

**Fig. 3:**
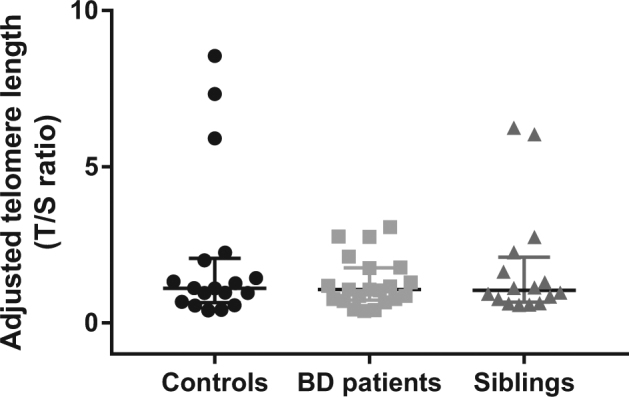
Telomere length in patients with BD, siblings, and healthy controls. The results are displayed as median ± 95% confidence interval. No significant differences were detected between groups (*p* > 0.05)

Contrary to our hypothesis, we did not find a significant correlation between telomere length and chronological age in our sample (*r*_s_ = –0.117, *p* = 0.394). We also found no statistical differences in telomere length between men and women (U = 269, *p* = 0.203). The groups did not show any significant differences with regard to the telomere length when controlling for chronological age, sex, and cell count estimates (*F*(2, 43) = 0.486, *p* = 0.619, and η^2^_p_ = 0.022, Fig. 3). Telomere length did not significantly correlate with epigenetic accelerated aging (*r*_s_ = 0.005, *p* = 0.971) or mtDNA copy number (*r*_s_ = 0.197, *p* = 0.148) either. These correlations remained to be not significant even when we split our sample into younger and older subjects (younger subjects—*F*(2, 18) = 0.692, *p* = 0.513, and η^2^_p_ = 0.071; older subjects—*F*(2, 14) = 1.222, *p* = 0.324, and η^2^_p_ = 0.149).

### Association with clinical parameters

Patients with BD presented significantly lower global functioning scores as assessed by the GAF scale compared to both controls and siblings (F(2, 47) = 71.382, *p* < 0.001, and η^2^_p_ = 0.752, Table 1). Based on this, we sought to investigate whether global functioning would be associated with the aging markers previously measured in our sample. GAF scores showed a positive and moderately strong correlation with mtDNA copy number (*r*_s_ = –0.364, *p* = 0.013), and a higher mtDNA copy number was associated with a greater number of episodes within the BD group (*r*_s_ = 0.353, *p* = 0.02). By contrast, GAF scores did not correlate with chronological age (*r* = 0.032, *p* = 802), epigenetic age acceleration (*r*_s_ = –0.024, *p* = 0.872), or telomere length (*r*_s_ = –0.017, *p* = 0.910). Telomere length did not show any significant correlations with clinical variables, including illness duration (*r* = –0.206, *p* = 0.383) or total number of episodes (*r* = 0.068, *p* = 0.777). Further correlations between mtDNA copy number and epigenetic age with other clinical variables (mood symptoms scores, age of illness onset, and length of illness) did not reach statistical significance (*p* > 0.05).

## Discussion

Given the dearth of findings on the role of accelerated biological aging in the pathophysiology and progression of complex psychiatric disorders, this study aimed to compare multiple biological clocks in BD patients, siblings, and healthy controls. The most compelling finding of this study is that patients displayed an accelerated epigenetic aging that seems to be at least partly associated with an increase in mtDNA copy number. Furthermore, both of these aging markers were independent of telomere shortening. To our knowledge, this is the first study to examine epigenetic aging and its association with other biological aging markers in BD.

The association between DNA methylation and aging has been suggested by several studies^48^, and so has the use of epigenetic clocks in predicting chronological age^24,49^. In line with previous results^34^, our current findings suggest that DNAm age might represent a better aging marker than the commonly used telomere length, particularly in older individuals. By being dynamically modulated, epigenetic aging has been shown to be influenced by a plethora of lifestyle factors^50^ and to accelerate with the cumulative effects of life stressors^25^. This might be one of the reasons why we only detected significant differences in epigenetic aging in older subjects, especially considering the key role proposed for chronic stress^51^ and the high rates of unhealthy lifestyles seen in BD patients^52,53^.

Our findings suggest that accelerated epigenetic aging in BD may be at least partially associated with an increase in mtDNA copy number. Studies specifically addressing mtDNA levels in BD show conflicting results. While some authors report no differences in patients^54–58^, others report both decreased^59^ and increased^60^ levels compared to controls. Although mtDNA copy number has been shown to decline with age in specific populations^28^, its content is thought to slightly increase in the fifth decade of life and later decline in older subjects^61^. Moreover, Tyrka and collaborators have recently shown that early life stress is associated with an increase in mtDNA copy number, which has been hypothesized as a compensatory mechanism to the aging-related increases in energy demand or reduced mitochondrial function^41^. A similar interpretation could be adopted for our findings based on the mitochondrial dysfunction consistently reported for BD patients^58,62^ and the strong association between BD and early life stress^63^.

To the best of our knowledge, this is the first study to suggest a link between mtDNA copy number and the epigenetic clock. Accordingly, previous studies have reported an important mitochondrial–nuclear cross talk and an association between DNA methylation and mtDNA^64,65^. Nuclear DNA methylation significantly interferes with mitochondrial function since most mitochondrial proteins are encoded by the nuclear genome. For instance, polymerase γ catalytic subunit, which is a mitochondrial protein encoded in the nucleus, is known to modulate mtDNA replication, and its methylation status has been negatively correlated with mtDNA copy number^66^. In particular, our exploratory analysis showed that 17 CpGs included in the clock CpGs used to predict DNAm age are actually located within nuclear-encoded mitochondrial genes. Even though this finding does not confirm mitochondrial function as a top mechanism related to the epigenetic clock, it does suggest an interesting loop of modulation between nuclear DNA methylation and mitochondrial function that might underlie the accelerated aging process observed in our sample.

Contrary to our hypothesis, we were unable to find a significant correlation between telomere length and chronological age in our sample. This suggests a nonlinear association between aging and telomere shortening, at least in specific populations. Accordingly, a recent study in a large cohort showed that telomere length declines over midlife but remains stable (and actually subtly increases) in later life^67^. In addition, some medications used for BD treatment have been shown to increase telomere length, including lithium^17,18^. Along this line, our sample was medicated and 23.8% took lithium. Finally, our results showing no differences in telomere length are consistent with a recent meta-analysis that suggests that telomere shortening is not seen in all BD samples^14^.

Our findings suggest independent mechanisms regulating the association between telomere length with the epigenetic clock and mtDNA copy number. In fact, telomere length and epigenetic clock have been previously shown to be uncorrelated and to independently predict chronological age^34,35^. Furthermore, some authors speculate that epigenetic aging and cellular senescence are independent mechanisms that do not necessarily happen together^35^. Indeed, DNAm age does not measure mitotic age or cellular senescence^24^ and has been hypothesized to contain information that is complementary to that of the telomere clock^35^. Of note, the lack of association between telomere length and mtDNA copy number in our sample contradicts recent evidence of a cross talk between both markers^31,33^. This finding may indicate the presence of mechanisms specific to our population of medicated BD patients.

There are several possible explanations for the accelerated aging seen in our sample of BD patients. So far, it is unclear whether such an accelerated epigenetic aging is specifically related to BD’s genetic makeup or to the direct effects of environmental parameters, such as general medical comorbidities, use of tobacco and alcohol, obesity, exposure to different medications, toxins, and lifetime-stressful events, among others. All of these could be at least partly linked to an increase in oxidative stress (as consistently reported in BD patients^68,69^), which might drive an aging acceleration through damage accumulation^70^. The lack of differences in siblings (individuals at familial risk for BD) suggests that genetic predisposition alone may not be enough to determine epigenetic accelerated aging, at least in our particular sample. The most likely hypothesis is that both genetic background and environmental exposure interact to determine such acceleration. Also, BD patients have been reported to present a dysfunctional HPA axis and therefore be less resilient to stress^71^, which could potentially feed a vicious cycle of stress-induced epigenetic aging acceleration.

Finally, contrary to our hypothesis, we did not find a statistically significant epigenetic aging acceleration in the cerebellum of patients with BD. In accordance with these results, a recent study found no evidence of accelerated brain aging in BD based on the analysis of aging-related imaging structural trajectories^72^. Nonetheless, a previous study assessing the epigenetic clock has shown that the cerebellum ages more slowly than other tissues possibly due to mechanisms involving RNA helicases^73^. Based on this, we cannot rule out the possibility that epigenetic acceleration may be detectable in other brain regions from patients with BD that are more tightly related to mood symptoms than the cerebellum, such as the prefrontal cortex or the hippocampus.

One of the limitations of this study is our small sample size, which precluded us from analyzing the effects of medications and other important demographic variables in our results, including the effects of comorbidities and the different diagnoses presented by some of the siblings included in the study. Also, the cross-sectional design of this study did not allow us to directly assess aging ‘acceleration’ *per se*, which will only be confirmed by longitudinal studies. Moreover, information on participants’ history of lifetime trauma exposure and early life stress was not available, which limited the analysis of stress influences on age acceleration in our sample. In this sense, childhood trauma and stress may play a role in the mechanisms regulating the association between telomere length and mtDNA copy number^41^, and have been associated with a shorter telomere length and disease risk^74^. Also, medication use and its heterogeneity among patients may represent potential important confounders in our results.

In summary, the findings of this study provide novel evidence of accelerated epigenetic aging in BD patients. We showed that accelerated epigenetic aging measured in peripheral blood is partly associated with a higher mtDNA copy number, but is independent of telomere shortening. Future longitudinal studies focused on the mechanisms underlying the link between accelerated aging and vulnerability to BD are warranted, as well as the assessment of aging markers in other tissues of relevance to the pathophysiology of BD. The complex interplay between different aging markers in BD highlighted in this study provides evidence that the epigenetic clock may be a relevant novel target for future treatment and prophylactic interventions for BD.

## Electronic supplementary material

Supplemental Information
