# Supplementary material for: Accelerated epigenetic aging and mitochondrial DNA copy number in bipolar disorder
Source: Transl Psychiatry. 2017 Dec 11;7:1283. doi: 10.1038/s41398-017-0048-8 (PMC5802567; doi:10.1038/s41398-017-0048-8)
Supplement: Supplementary file 1 — Supplemental Information [file 41398_2017_48_MOESM1_ESM.docx]

**Supplementary Methods**

*Genome-wide methylation analysis*

Preprocessing of data included filtering out probes outside of CpG context, probes overlapping with SNPs, located on sex chromosomes, and probes of highest impurity based on their detection *p*-value. Background was subtracted using the *methylumi* package and signal intensity values were normalized using the SWAN normalization method, as implemented in the *minfi* package. Differentially methylated probes were assessed using hierarchical linear models from the *limma* package, which were employed and fitted using an empirical Bayes approach on derived M-values. Multiple testing corrections were applied using Benjamini and Hochberg procedure to control for false discovery rate (FDR). We also used the function estimateCellCounts() on the package *FlowSorted.Blood.450k* in *minfi* to estimate the cell type composition of blood by using a modified version of the algorithm described by Houseman et al. (1).

*Mitochondrial (mt) DNA copy number*

Real-time quantitative polymerase chain reactions (PCRs) were performed to measure the amount of mitochondrial DNA relative to a single-copy gene (beta-hemoglobin) with a modified protocol from Tyrka and collaborators (2). Reactions included 25 ng genomic DNA, 300 nmol/L of each primer, and 1 x Sybr Select Master Mix (Life Technologies, Carlsbad, CA, USA) in a final volume of 10 μl. Primer sequences and PCR cycling conditions for both mtDNA and beta-hemoglobin have been previously reported (2). Reactions were carried out in 96-well plates and data were acquired in a QuantStudio™ 7 Flex Real-Time PCR System (Life Technologies). mtDNA copy number for each sample was determined by relative quantification based on a 5-point standard curve performed with a serial dilution (1:2) of a calibrator sample ranging from 1 to 0.0625 ng DNA. All samples were analyzed in triplicate. The relative amount of mtDNA was finally divided by the relative amount of the beta-hemoglobin gene to obtain an index of mitochondrial DNA copy number.

*Telomere length*

Telomere length was measured by singleplex real-time quantitative PCR as previously described (3), with minor modifications. The relative telomere length (T/S) was estimated by quantifying the levels of telomere (T) and β-globin (HBG), which was taken as a single copy gene (S). Relative telomere length was calculated for each sample by comparison to a 5-point standard curve derived from the amplification, in the same plate, of serial dilutions of a calibrator DNA sample (1:1.68). Primer sequences and PCR cycling conditions have been previously reported (3). All reactions were run in duplicate in 96-well plates and included 15mM Tris pH 8.0, 50mM KCl, 2mM MgCl_2_, 0.2mM deoxynucleotide (dNTP) mix (ThermoFisher Scientific, Waltham, MA, USA), 5mM dithiothreitol, 1% dimethyl sulfoxide, 0.375 U AmpliTaq Gold DNA polymerase (ThermoFisher Scientific), 150 nM 6-ROX (C6156, ThermoFisher Scientific), 0.2 x SYBR® Green I (ThermoFisher Scientific), and 21 ng genomic DNA. The final primer concentrations were: 270 nM for tel1, 900 nM for tel 2, 400 nM for HBG1, and 400 nM for HBG2. Data were acquired in a QuantStudio™ 7 Flex Real-Time PCR System (Life Technologies), and specificity of the amplifications was confirmed at the end of each run using melting curve analysis.


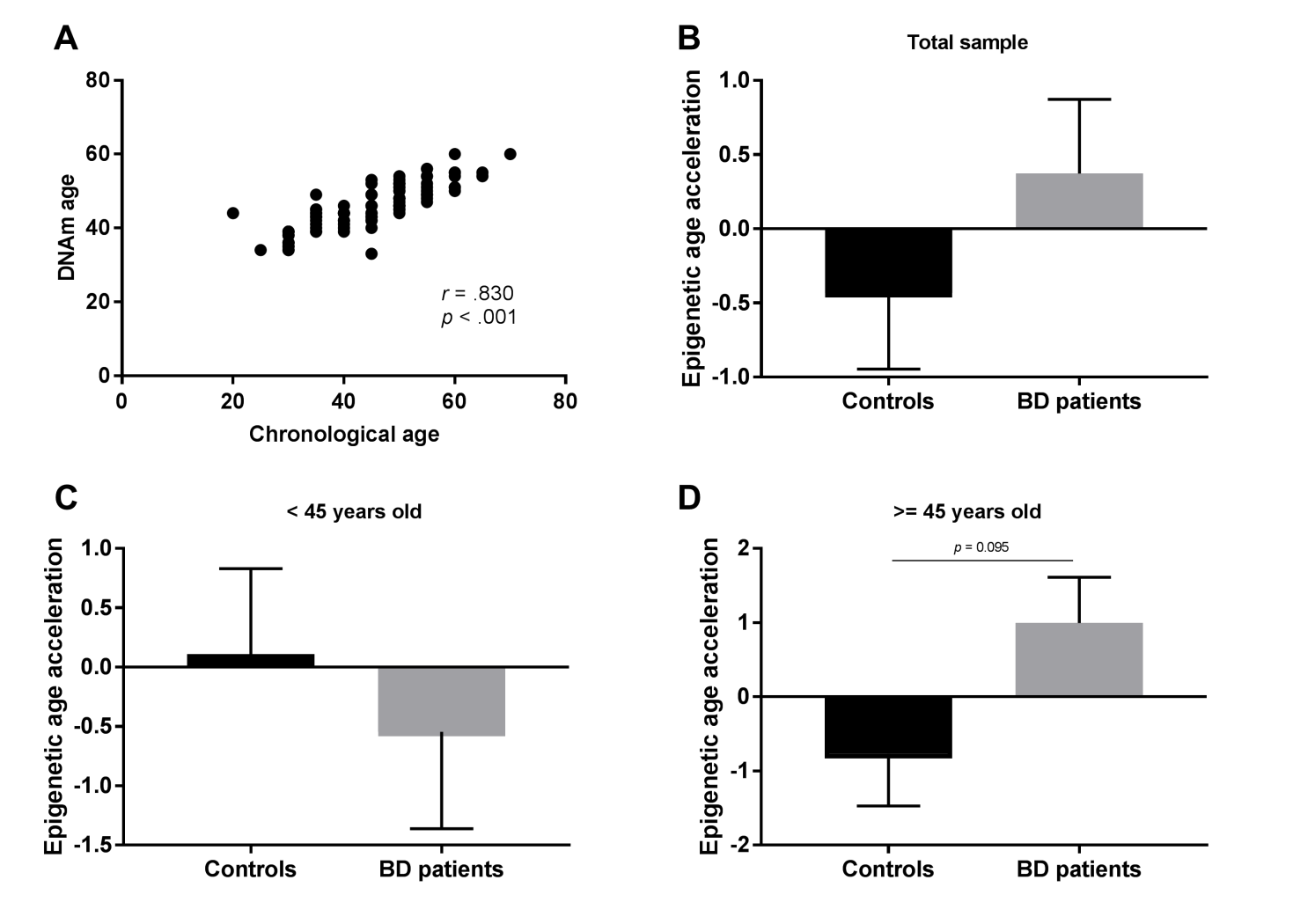


**Figure S1.** Epigenetic clock in postmortem cerebellum tissue from patients with bipolar disorder and non-psychiatric controls. Genome-wide DNA methylation (DNAm) data were obtained from a publicly available dataset (GSE38873) and analyzed with the Horvath algorithm for prediction of DNAm age. A) Predicted DNAm age and chronological age show a strong and significant positive correlation. B) Epigenetic age acceleration was estimated by regressing the DNAm age on chronological age. The residuals from the regression were taken as a measure of age acceleration and compared between groups using general linear models with post-mortem interval as a co-variate. When comparing controls (n = 47) versus patients (n = 47), no statistically significant difference was found in epigenetic age acceleration (*p* = 0.177). When splitting the sample into younger (C) and older (D) subjects based on the median age of the total sample (45 years old), no significant difference between patients (n = 19) and controls (n = 18) was detected among the younger subjects (*p* = 0.554), nor between patients (n = 28) and controls (n = 29) among the older ones (*p* = 0.095).

**Table S1.** Cell count estimates.

| **Cell type** | **Controls** | **BD I** | **Siblings** | ***P*-value** |
| --- | --- | --- | --- | --- |
| B-lymphocytes, median (IQR) | 0.049 (0.02) | 0.055 (0.03) | 0.066 (0.05) | .089^a^ |
| Granulocytes, median (IQR) | 0.625 (0.08) | 0.589 (0.14) | 0.603 (0.12) | .602^a^ |
| Monocytes, mean ± SD | 0.068 ± 0.02 | 0.083 ± 0.03 | 0.092 ± 0.02 | .154^b^ |
| Natural killer cells, median (IQR) | 0.068 (0.08) | 0.055 (0.06) | 0.061 (0.04) | .908^a^ |
| CD4+ T-lymphocytes, mean ± SD | 0.132 ± 0.06 | 0.140 ± 0.06 | 0.143 ± 0.07 | .914^b^ |
| CD8+ T-lymphocytes, median (IQR) | 0.060 (0.04) | 0.046 (0.06) | 0.048 (0.07) | .578^a^ |

Legend: IQR – interquartile range. ^a^Kruskall-Wallis test; ^b^one-way ANOVA.

**Table S2**. Postmortem tissue demographics (GSE38873)

|  | **Non-psychiatric controls (n = 47)** | **BD patients (n = 47)** | ***P*-value** |
| --- | --- | --- | --- |
| Age (years), mean ± SD | 45.11 ± 9.1 | 44.47 ± 11.4 | .766^a^ |
| DNAm age (years), mean ± SD | 45.56 ± 5.3 | 46.07 ± 6.9 | .696^a^ |
| PMI, median (IQR) | 28 (14) | 32 (25) | .055^b^ |

Legend: BD – bipolar disorder; DNAm – DNA methylation; F – female; IQR – interquartile range; M – male; PMI – postmortem interval. ^a^independent t-test; ^b^Mann-Whitney test.

**Table S3**. Nuclear-encoded mitochondrial genes among the 353 clock CpGs

| **Gene** | **Definition** | **Location** | **Probe** | **Correlation coefficient with age (Horvath, 2013)** | **Correlation coefficient with age (current sample)** |
| --- | --- | --- | --- | --- | --- |
| *ACOX1* | acyl-CoA oxidase 1, palmitoyl | 17q25.1 | cg16419345 | 0.282563693 | -0.092 |
| *ADHFE1* | alcohol dehydrogenase, iron containing, 1 | 8q13.1 | cg08090772 | -0.341458098 | 0.050 |
| *ALKBH3* | alkB, alkylation repair homolog 3 (E. coli) | 11p11.2 | cg22637507 | -0.161364515 | 0.093 |
| *APOA1BP* | apolipoprotein A-I binding protein | 1q22 | cg16168311 | -0.196860712 | -0.135 |
| *ATPAF1* | ATP synthase mitochondrial F1 complex assembly factor 1 | 1p33 | cg23786576 | -0.359681997 | -0.063 |
| *ELAC2* | elaC ribonuclease Z 2 | 17p12 | cg26162695 | -0.043863368 | 0.084 |
| *EPHX2* | epoxide hydrolase 2, cytoplasmic | 8p21.2-p21.1 | cg24081819 | -0.00950238 | -0.178 |
| *FXN* | frataxin | 9q21.11 | cg07158339 | -0.626329239 | -0.476 |
| *HTRA1* | HtrA serine peptidase 2 | 10q26.13 | cg02154074 | 0.044757003 | 0.364 |
| *MRPL38* | mitochondrial ribosomal protein L38 | 17q25.1 | cg11653266 | -0.176347192 | 0.148 |
| *MRPS21* | mitochondrial ribosomal protein S21 | 1q21.2 | cg18031008 | 0.264553633 | 0.119 |
| *NDUFA13* | NADH dehydrogenase (ubiquinone) 1 alpha subcomplex, 13 | 19p13.11 | cg21395782 | -0.123872644 | 0.072 |
| *NDUFA3* | NADH dehydrogenase (ubiquinone) 1 alpha subcomplex, 3, 9kDa | 19q13.42 | cg07455279 | 0.005632264 | 0.077 |
| *NDUFS5* | NADH dehydrogenase (ubiquinone) Fe-S protein 5, 15kDa (NADH-coenzyme Q reductase) | 1p34.3 | cg07388493 | -0.520727113 | -0.450 |
| *NT5DC3* | 5'-nucleotidase domain containing 3 | 12q23.3 | cg06462291 | 0.015758987 | 0.122 |
| *PDK4* | pyruvate dehydrogenase kinase, isozyme 4 | 7q21.3 | cg22171829 | -0.133887348 | -0.333 |
| *TIMM17A* | translocase of inner mitochondrial membrane 17 homolog A (yeast) | 1q32.1 | cg15262928 | -0.078482669 | 0.050 |

**References**

1. Houseman EA, Accomando WP, Koestler DC, Christensen BC, Marsit CJ, Nelson HH, et al. (2012): DNA methylation arrays as surrogate measures of cell mixture distribution. *BMC bioinformatics*. 13:86.

2. Tyrka AR, Parade SH, Price LH, Kao HT, Porton B, Philip NS, et al. (2016): Alterations of Mitochondrial DNA Copy Number and Telomere Length With Early Adversity and Psychopathology. *Biological psychiatry*. 79:78-86.

3. Cawthon RM (2002): Telomere measurement by quantitative PCR. *Nucleic acids research*. 30:e47.
